# Identification of novel genetic variants predisposing to familial oral squamous cell carcinomas

**DOI:** 10.1038/s41421-019-0126-6

**Published:** 2019-11-26

**Authors:** Yaping Huang, Jizhi Zhao, Guogen Mao, Grace Sanghee Lee, Jia Zhang, Lijun Bi, Liya Gu, Zhijie Chang, Joseph Valentino, Guo-Min Li

**Affiliations:** 1 Department of Radiation Oncology, University of Texas Southwestern Medical Center, Dallas, TX 75390 USA; 2 Department of Toxicology and Cancer Biology, University of Kentucky College of Medicine, Lexington, KY 40536 USA; 3 Department of Otolaryngology, Head & Neck Surgery, University of Kentucky College of Medicine, Lexington, KY 40536 USA; 4 Department of Stomatology, Peking Union Medical College Hospital, Beijing, 100730 China; 5 Insititute of Biophysics, Chinese Academy of Sciences, Beijing, 100101 China; 6 Department of Basic Medical Sciences, Tsinghua University School of Medicine, Beijing, 100084 China

**Keywords:** Cancer genetics, Oral cancer, Oncogenes

## Abstract

Oral squamous cell carcinoma (OSCC) is a common subtype of head and neck squamous cell carcinoma (HNSCC), but the pathogenesis underlying familial OSCCs is unknown. Here, we analyzed whole-genome sequences of a family with autosomal dominant expression of oral tongue cancer and identified proto-oncogenes *VAV2* and *IQGAP1* as the primary factors responsible for oral cancer in the family. These two genes are also frequently mutated in sporadic OSCCs and HNSCCs. Functional analysis revealed that the detrimental variants target tumorigenesis-associated pathways, thus confirming that these novel genetic variants help to establish a predisposition to familial OSCC.

## Introduction

Oral squamous cell carcinoma (OSCC) is one of the most common cancer types worldwide and occurs frequently in Western countries^1,2^. In the United States, more than 50,000 OSCC cases were diagnosed in 2018, with more than 10,000 deaths. Tobacco use and alcohol consumption are considered the major risk factors for OSCCs^3–5^. Human papillomavirus (HPV) is another risk factor^6,7^. Although OSCCs occur sporadically in populations, epidemiological studies have suggested hereditary risks for OSCCs^8,9^. However, the hereditary factors that predispose to OSCCs are largely unknown.

We have encountered a family with autosomal dominant expression of oral tongue cancer. The index case presented with diffuse carcinoma in situ of the oral tongue in her sixth decade. She was treated with multiple surgical excisions, radiation therapy and chemotherapy. She died of the disease later. All three of her children (two sons and a daughter) have manifested similar disease, always on the oral tongue. Her elder son and daughter died of the disease in their early 60’s, and the second son has undergone two resections of premalignant tumors of the oral tongue. To the best of our knowledge, none of these individuals used tobacco, and none heavily used alcohol. We therefore believe that the family’s oral tongue cancer is associated with a hereditary genetic factor.

To identify the genetic defect responsible for the disease, we performed whole genome sequencing (WGS) of available genomic DNA from the family and identified *VAV2* and *IQGAP1* as the primary causative factors for individual family members. Targeted sequencing analysis revealed frequent mutations of these two genes in sporadic OSCCs and cell lines. These genes also showed much higher mutation frequencies in HNSCCs than in any other cancer types documented in the International Cancer Genome Consortium (ICGC) dataset. Protein structure and functional analyses indicated that the genetic variants identified in the family with oral cancer alter the functions of the proteins, affecting several important tumorigenesis-associated pathways, such as the MAPK and PI3K/AKT pathways (see the flow chart of the whole study in Supplementary Fig. S1). Therefore, our study provides new insights into the genetic factors underlying a family history of OSCCs and the pathways by which OSCCs develop.

## Results

### Identifying familial OSCC susceptible variants

**Fig. 1:**
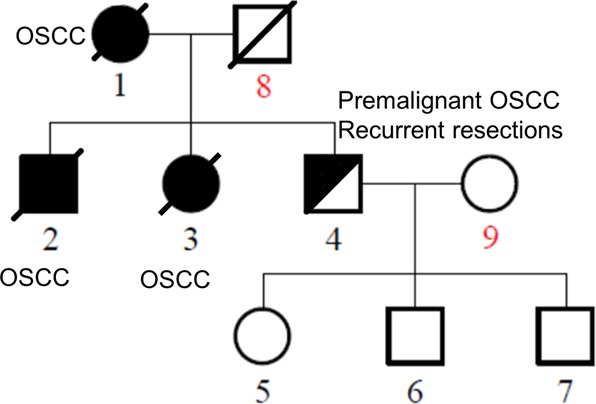
Pedigree chart of the family with OSCCs in the “discovery set”. Family members are shown and numbered. Filled symbol, affected; half-filled symbol, recurrent premalignant tumors; oblique line, deceased. The family members diagnosed with OSCC are indicated in the figure

To determine genetic factors contributing to familial OSCCs, we performed whole genome sequencing analysis of seven members of a family with OSCC (Fig. 1: cases 1, 3, 4–7, 9). As shown in Fig. 1, three family members (cases 1–3) had oral tongue cancer and died of the disease (also see Supplementary Table S1); a fourth member (case 4) has premalignant oral tongue tumors. Case 9 (SF-002S) is a healthy individual in the family. Since she does not share any inherited genetic material with cases 1–4 and is the mother of cases 5–7, she serves as an excellent control in this study. The average sequencing depth of each sample is ~40×, except for case 1 (SF-001M), which is about 27× depth. Genomic loci with more than 8 × depth cover about 99% of the whole genome in all the samples (Supplementary Table S2), indicating that the sequencing data can be trusted for identifying variations.

After applying standard quality control procedures, we mapped sequencing reads to the human reference genome (UCSC hg19) with BWA and GATK programs^10,11^, and comprehensive variant calling was performed by some in-house pipelines (Materials and Methods). Subsequently, all variants were annotated by ANNOVAR^12^. On average, 3.7 million single nucleotide variations (SNVs), 0.6 million small insertions and deletions (InDels), 1000 copy number variations (CNVs), and 800 structure variations (SVs) were identified in each sample (Supplementary Tables S3–6).

We selected the variants with high quality that are also found in the HapMap dataset for linkage analysis of the sequencing data. This analysis confirmed the pedigree relationships for all family members. Linkage regions with positive logarithm of odds (LOD) scores, which is equal to log_10_ (odds ratio), were retained as candidate inherited regions. To identify OSCC susceptibility variants, we excluded the common variants (variant allele frequency > 0.5%) annotated in public databases including HapMap^13^, 1000 Genomes Project^14^, and dbSNP^15^. As a result, only non-synonymous and splicing-site variants were retained for subsequent filtration. In the absence of the third generation (cases 5–7) information and the inheritance mode, we performed different combinations of analyses under both dominant and recessive modes, followed by functional prediction analysis using SIFT^16^ and polyphen2^17^. Variants predicted to be “Damaging” were retained. We then conducted haplotype analysis and sequence conservation estimation to identify variants with high sequence conservation scores that are associated with all OSCC cases, but not with case 9, who did not inherit any genetic material from the family. As shown in Table 1, eight SNVs affecting eight genes and one InDel affecting another gene met these stringent filtration conditions (Supplementary Table S7) and were verified by Sanger sequencing. Sequence conservation analysis by three different datasets or methods indicated that all these variants are relatively conserved across different species (Supplementary Fig. S2), implying their important roles during evolution.

**Table 1:** Summary of the genetic mutations in the familial OSCCs

Gene	Genomic position	Transcript ID	Nucleotide mutation	Amino acid change	Protein domain	Sanger sequence confirmation	Gene description
*IQGAP1*	Chr15:90996413	ENST00000268182.5	c.1376 C>T	p.Ser459Leu	IR-WW domain	Yes	IQ motif containing GTPase activating protein 1
*VAV2*	Chr9:136629207	ENST00000371850.3	c.2614 G>A	p.Val872Ile	SH3 domain	Yes	Vav guanine nucleotide exchange factor 2
*DCN*	Chr12:91552082	ENST00000052754.5	c.529 A>T	p.Ile177Phe	LRR 5	Yes	Decorin
*TBC1D10C*	Chr11:67176983	ENST00000312390.5	c.1099 C>T	p.Arg367Cys	None	Yes	TBC1 domain family member 10C
*PCDHGC5*	Chr5:140869229	ENST00000252087.1	c.422 G >A	p.Arg141His	Cadherin 2 domain	Yes	Protocadherin gamma subfamily C, 5
*SLC7A8*	Chr14:23598870	ENST00000316902.7	c.1252 C>T	p.Arg418Cys	None	Yes	Solute carrier family 7 member 8
*SLC14A2*	Chr18:43247837	ENST00000255226.6	c.1757G>A	p.Arg586Gln	None	Yes	Solute carrier family 14 member 2
*KIAA0556*	Chr16:27642442	ENST00000261588.4	c.367 C>T	p.Arg123Trp	None	Yes	–
*PYROXD2*	Chr10:100167689	ENST00000370575.4	c.212delG	p.Gly71fs	FAD dependent oxidoreductase	Yes	Pyridine nucleotide-disulfide oxidoreductase domain 2

### The susceptibility genes *VAV2* and *IQGAP1* are highly mutated in sporadic oral cancers and cell lines

**Fig. 2:**
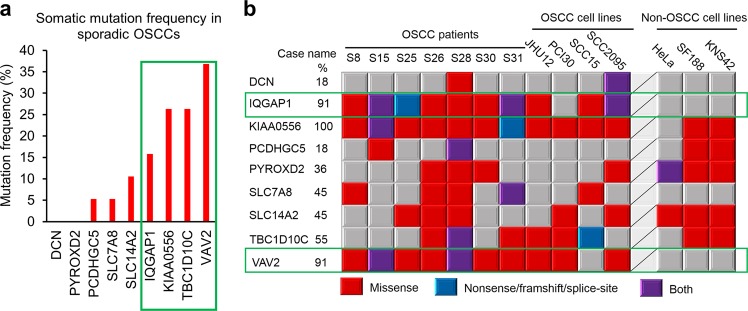
Mutation frequencies of the familial OSCC predisposition genes in sporadic OSCC cases and cell lines. **a** Bar graph of the somatic mutation frequencies (percentage of cases with detrimental mutations) of the nine genes in 19 normal-tumor paired cases. **b** Mutation landscape of the nine genes in seven tumor tissues without paired normal tissue controls, four oral cancer cell lines and three non-oral cancer cell lines. Gene symbols and percentage of mutations in OSCC patients and OSCC cell lines are shown on the left. Mutation types are indicated on the bottom

To further confirm that the identified variant genes are indeed susceptible to developing OSCC, we performed targeted massively parallel sequencing of the coding regions of the 9 identified genes in 26 oral tongue tumors (19 of which were paired tumors and their adjacent normal tissues, see Supplementary Table S8) and 4 oral cancer and 3 non-oral cancer cell lines. Among the 19 pairs of normal-tumor cases, the somatic mutation frequencies of these genes ranged from 5.3 to 36.8%, with *VAV2*, *TBC1D10C*, *KIAA0556*, and *IQGAP1* showing the highest mutation frequencies (Fig. 2a, green box). However, no detrimental somatic mutations were identified in *DCN* and *PYROXD2* (Fig. 2a). Similarly, *VAV2* (91%), *IQGAP1* (91%), and *KIAA0556* (100%) were the most frequently mutated genes in the oral cancer cell lines JHU12, PC130, SCC15, and SCC2095, as well as in seven sporadic oral cancer cases without paired normal tissue controls (Fig. 2b). However, when we compared with sequencing data from the three non-OSCC cell lines, HeLa (cervical carcinoma), SF188, and KNS42 (pediatric gliomas), we found that only mutations in *VAV2* and *IQGAP1* genes seemed to be specific to oral cancers, as all three non-oral cancer lines displayed mutations in the other genes, but not in *VAV2* and *IQGAP1* (Fig. 2b, green boxes). Importantly, we noted that some of the mutations in the sporadic cases and cell lines are located in the same domain or even the same amino acid residue as the susceptibility variants identified in the family members, e.g., Ser458Leu mutation of IQGAP1 in the SCC15 cell line and Val872Ile substitution of VAV2 in case S31. Taken together, these results imply that *VAV2* and *IQGAP1* are indeed associated with OSCCs.

### *VAV2* and *IQGAP1* are preferentially mutated in head and neck squamous cell carcinomas

**Fig. 3:**
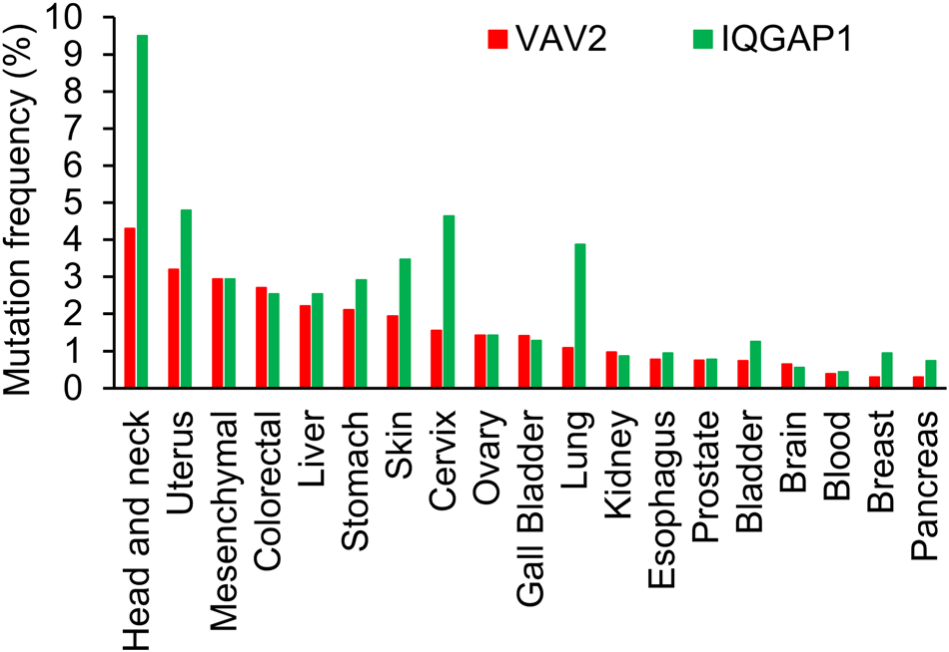
Mutation frequencies of *VAV2* and *IQGAP1* in different cancer types. Cancer types were categorized by anatomic sites. Mutation data were extracted from the ICGC dataset. Mutation frequency was calculated as the percentage of cases with mutations in the target genes

Oral tongue cancer is a subtype of head and neck squamous cell carcinoma (HNSCC). Previous studies have identified several driver mutations in HNSCC, including *TP53*, *CDKN2A*, *PTEN*, *PI3CA*, *HRAS*, and *NOTCH1*^5,18–20^. Surprisingly, we did not find any of these previously identified genes but a new set of genes affecting all the oral cancer cases in the family. To determine if the susceptibility genes identified in the OSCC family are associated with HNSCCs, we screened the ICGC dataset and analyzed the mutation frequencies of *VAV2* and *IQGAP1* in different cancer tissues derived from 19 different anatomic sites. The results revealed that the mutation frequencies of both *VAV2* and *IQGAP1* are higher in HNSCCs than in any other cancer types (Fig. 3). These results indicate that *VAV2* and *IQGAP1*, identified in a family with OSCC, are specific genetic factors that predispose to HNSCCs, including OSCC.

### Functional assessment of the susceptibility variants in *VAV2* and *IQGAP1*

A number of common and cancer type-specific driver pathways have been identified, including the TGFB, EGF, Notch, and HIF-1a pathways^21^. However, little is known about the pathogenic pathway of OSCCs, especially familial OSCCs. Thus, we performed KEGG pathway analysis on the candidate genes and found that *IQGAP1* and *VAV2* were both enriched in the “Proteoglycans in Cancer” pathway (hsa05205, Supplementary Fig. S3). These two genes are on the upstream of this pathway, which regulates cell survival, proliferation, migration, and apoptosis. The data therefore suggest that *VAV2* and *IQGAP1* play important roles in tumorigenesis.

To further investigate the potential roles of *VAV2* and *IQGAP1* in OSCC development, we performed protein–protein interaction (PPi) network analysis of the two proteins by Cytoscape2.0^22^. Only proteins that directly interact with the candidate proteins are shown in the network. We found that the interaction clusters of IQGAP1 and VAV2 are linked together through RAC1, and they also interact with several important oncogenes and tumor suppressors involved in tumorigenesis (Supplementary Fig. S4). These findings are consistent with the pathway analysis and further demonstrate that VAV2 and IQGAP1 promote familial OSCC development.

We performed KEGG pathway enrichment analysis of the genes involved in the PPi network (Supplementary Fig. S4) to identify the pathways that these genes frequently and widely influence. We found that three pathways, “Pathways in cancer” (hsa05200), “Focal adhesion” (hsa04510), and “Insulin signaling pathway” (hsa04910), are widely affected by IQGAP1 and VAV2 (Table 2). These results are consistent with the clinical phenotypes of the family members with both oral tongue cancer and type I diabetes.

**Table 2:** Significantly enriched pathways of the genes in the PPi network

KEGG ID	Description	Genes involved
hsa05200	Pathways in cancer	FN1/CDC42, MAPK1, CDH1, CTNNB1, APC/EGFR, GRB2, CBL, RAC1, RHOA
hsa04510	Focal adhesion	FN1/CDC42, MAPK1, KDR, CTNNB1/EGFR, GRB2, RAC1, RHOA, VAV2
hsa04910	Insulin signaling pathway	IRS1/MAPK1, PRKACA, CALM1/GRB2, SOCS1, CBL

**Fig. 4:**
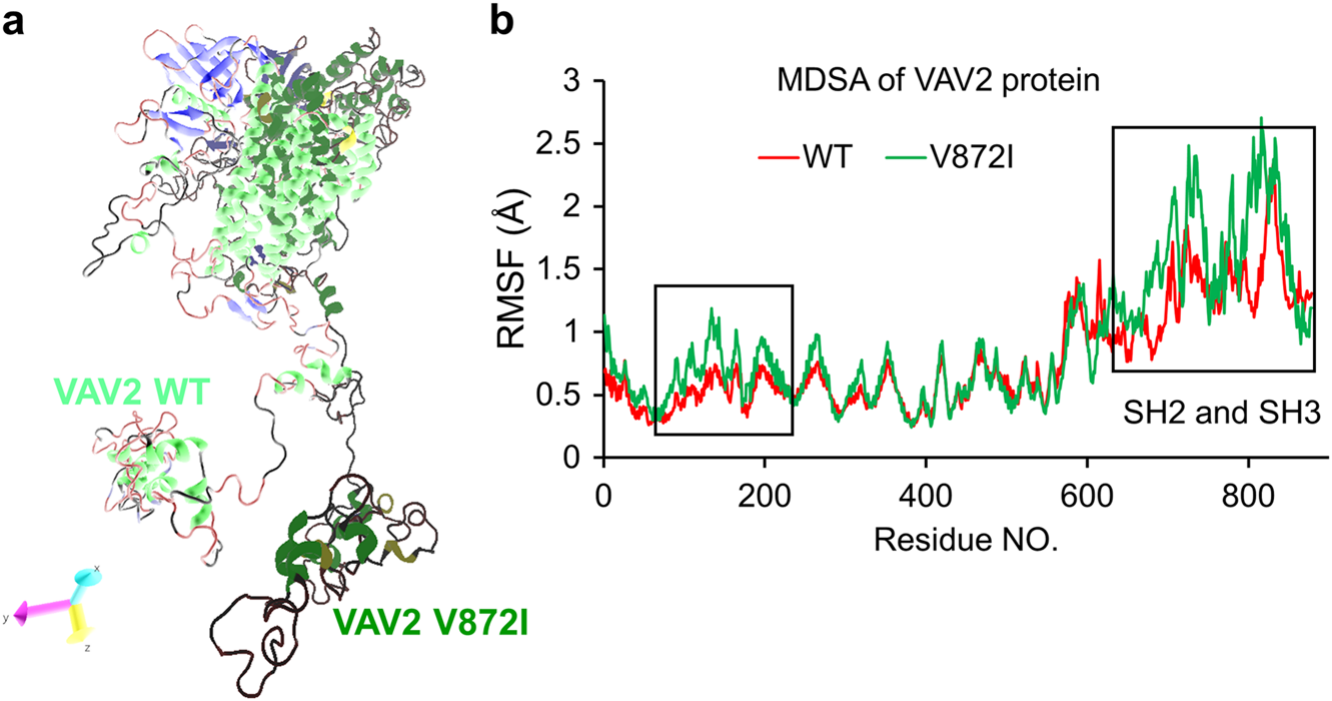
Modeling of VAV2 mutations by MDSA analysis. **a** Merged representative conformations of wild type and mutant VAV2 generated by 20 ns Molecular Dynamics Simulation Analysis (MDSA). **b** Residue-specific root mean square fluctuations (RMSF) in simulations. V872I mutation in VAV2 leads to instability of the whole protein, especially the N-terminal and the C-terminal SH2/3 domains, as shown in the black boxes

To further assess whether the variants in VAV2 and IQGAP1 alter the protein structures, we analyzed the conservation of the affected residues in these two proteins. We found that the Val872 and Ser459 residues are highly conserved across species (Supplementary Fig. S5). To test whether these mutations interfere with the protein structures and/or interactions with other proteins, we performed molecular dynamics simulation analysis (MDSA) of VAV2, whose structure model and size were available for this analysis. VAV2 works as a guanine nucleotide exchange factor (GEF) for the Rho/Rac family of GTPases and functions in a GEF-independent manner to organize the cytoskeleton^23^. VAV proteins that harbor mutations in the N-terminal CH domain, the CH domain plus the Ac region, and the PH domain show constitutive exchange activity and high transforming activity, which promotes tumor development^24,25^. The mutation we identified here is in the SH3 domain, which is a conserved domain that interacts with proteins and is involved in signaling pathways^26^. Thus, we built the structure model according to its interaction with SH3BP2, an SH3 domain binding protein. Given that Val872 is highly conserved across vetebrates (Supplementary Fig. S5a), we predicted that this mutation would alter the protein structure. MDSA analysis revealed that this was indeed the case (Fig. 4a, b). The V872I mutation leads to instability in both the N-ternimal and the C-terminal of VAV2, implying that the variation changes the normal protein functions by altering its structure and interaction networks, which further promotes tumorigenesis.

**Fig. 5:**
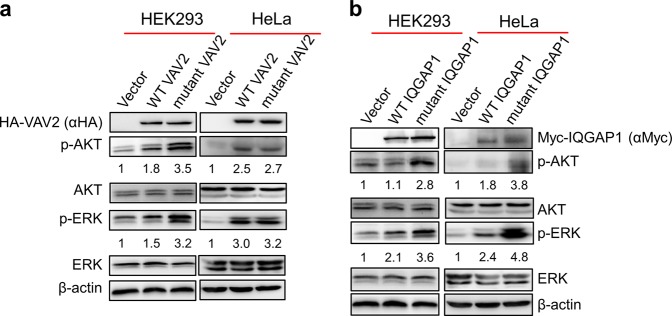
VAV2 and IQGAP1 mutants stimulate the MAPK and PI3K/AKT signaling pathways. Both WT and mutant HA-tagged VAV2 (**a**) and myc-tagged IQGAP1 (**b**) were expressed in HEK293 and HeLa cells, as indicated. The activation status of MAPK and PI3K/AKT pathways were determined by detecting the levels of phosphorylated ERK (p-ERK) and AKT (p-AKT). The relative fold change of p-ERK and p-AKT in the VAV2- and IQGAP1-expressed cells to that in the cells expressing empty vectors were calculated by normalizing to the total ERK and AKT expression levels respectively

To explore the possibility that the identified VAV2 and IQGAP1 variants promote tumorigesis, we ectopically expressed wild type (WT) and mutant VAV2 or IQGAP1 in HEK293 and HeLa cells, and analyzed the activation of the MAPK/ERK and PI3K-AKT/mTOR signaling pathways, both of which play critical roles in tumorigenesis^27,28^. As shown in Fig. 5, both AKT and ERK were activated in cells over-expressing either WT or mutated VAV2 (Fig. 5a) and IQGAP1 (Fig. 5b), consistent with the oncogenic activities of VAV2 and IQGAP1. However, AKT/ERK activation is elevated by about twofold in HEK293 cells expressing the muated VAV2 and IQGAP1 as compared with those expressing their corresponding WT proteins. Taken together, these results strongly suggest that the mutations associated with the oral cancer family stimulate the oncogenic activity of VAV2 and IQGAP1.

## Discussion

In this study, we identified *VAV2* and *IQGAP1* as the genetic basis of a case of oral cancer in a family. Surprisingly, these two genes are not among the driving factors identified previously in HNSCCs and OSCCs^5,18–20^, indicating that they are new oncogenic factors for OSCCs. Both genes exhibited heterozygous mutations and were inherited in an autosomal dominant fashion in the family with oral cancer. However, the only one family in this study may limit the implications of the discovery as it may not apply to the other cases. To verify the findings, we also analyzed 19 sporadic OSCCs for somatic mutations in *VAV2* and *IQGAP1* and found 8 of these sporadic cases carrying heterozygous alterations of these two genes (Fig. 2a). In addition, these genes are more frequently mutated in HNSCCs compared with tumors from other anatomic sites (Fig. 3). These observations suggest that the identified mutations in *VAV2* and *IQGAP1* are dominant drivers for both hereditary and sporadic OSCCs. We also noted that some of the previously identified OSCC genes^19,20^ are also mutated in certain members of the oral cancer family, but they are not as dominantly penetrated as *IQGAP1* and *VAV2* in the family. Given the heterogeneity and complexity of oral cancers developed from different histological subtypes, carcinogen exposure and molecular backgrounds^29,30^, further studies are required to comprehensively study the oncogenic epidemiology of both familial and sporadic OSCCs.

We provide evidence that both *VAV2* and *IQGAP1* have oncogenic potential. The identified VAV2 and IQGAP1 mutations appear to active their oncogenic activity, as these mutations activate the MAPK and PI3K/AKT pathways (Fig. 5). VAV2, a guanine nucleotide exchange factor, promotes oncogenesis through tyrosine kinase activity associated with the Rho/Rac/Ras-associated pathways^23^. N-terminal truncation or mutations of VAV2 induce constitutive activation of the protein through tyrosine phosphorylation^24,25,31^, resembling the mechanism of EGFR activation^32,33^. Unlike the previously identified mutations, which are located in the N-terminus of VAV2, the VAV2 mutation associated with the oral cancer family resides in the C-terminus of the protein. Even though the mutation is a single amino acid substitution, it appears to have altered the structure of VAV2 (Fig. 4). It is possible that the mutation stabilizes the protein, possibly by altering interactions with other factors in the VAV2-Rac1/cdc42/Rho signaling pathway via the SH2/3 domains (Fig. 4b). As such, the single amino acid substitution may have elevated the oncogenic activity of VAV2 or rendered the mutated VAV2 to gain a new oncogenic function. There are many such molecules, and a good example is the *KRAS* gene, which encodes a small GTPase and is the most common locus for gain-of-function mutations and/or activation of oncogenic activity in human cancers by a single amino acid substitution^34,35^.

IQGAP1 acts as a scaffold to modulate important signal transduction in PI3K/mTOR/AKT and MAPK pathways and is highly associated with cancers, including HNSCC^36–38^. As an oncogene, IQGAP1 is overexpressed in certain cancers through gene amplification^36^. The heterozygous *IQGAP1* mutation associated with the family in this study is located in the IQGAP1-repeats (IR) and the tryptophan (WW) repeats (IR-WW) motif, which mediates IQGAP1 interactions with signal molecules such as Septins, mTORC1 and Akt^39^, thus this mutation may also lead to oncogenic activation and/or represents the gain of a new oncogenic function.

It is possible that only *VAV2* or *IQGAP1* mutation is responsible for the development of oral cancer in the family. However, the close linkage of both genes with the individuals in the family made it difficult to eliminate either one. Given the disease’s strong penetration in the family and the oncogenic activities of both factors, we believe that both genes are associated with the familial disorder. This idea seems to be supported by the fact that IQGAP1 and VAV2 are linked together in the same interaction clusters through RAC1 (Supplementary Fig. S4). However, the detailed mechanism by which these two factors promote oral cancer development remains to be investigated. It is worth mentioning that both VAV2 and IQGAP1 have also been implicated in diabetes^39,40^ (also see Table 2), a disorder that is co-associated with the family, particularly in cases 1, 4, and 5 (Fig. 1).

We also identified some big chromosomal alterations, including structural variations (SVs) in two big regions (Supplementary Table S9) and copy number variations (CNVs) in eight smaller regions (Supplementary Table S10). However, almost all these big chromosome abnormities occurred in the noncoding region and had little influence on intact protein-coding genes, indicating that these chromosome abnormities may not be the *cis*-genetic factors for OSCCs. Another factor closely associated with the family we studied is *DCN* (Table 1). We only eliminated this gene because we found no DCN mutations in the sporadic oral cancers examined in the study. Future study will provide further information to determine if DCN mutations and the big chromosomal alterations contribute to OSCCs.

## Materials and methods

### Sequencing samples

The “discovery set” consisted of samples from seven patients from three generations of an American family: SF-002P (61 years old, recurrent premalignant oral cavity tumors and resections), his mother and sister (SF-001M and SF-002G, recurrent oral squamous cell carcinoma patients, both deceased), his daughter (SF-003S, 22 years old, tumor free) and twin sons (SF-003C and SF-003J, 26 years old, tumor free), and his wife (SF-002S, tumor free), who served as a genetic background control (Fig. 1, Supplementary Table S1). Among them, SF-002P and his daughter were both diagnosed with type I diabetes. SF-001M also had type I diabetes. None of the family members used tobacco or heavily used alcohol. Paraffin-embedded tissues or blood samples were collected at the University of Kentucky with informed consent from the family members.

The “validation set” consisted of samples from 19 pairs of sporadic oral tumor-normal cases, 7 oral tumor cases only (Supplementary Table S8) and 4 oral cancer cell lines (PCI30, JHU12, SCC15, and SCC2095). Formalin-fixed tissues were collected from Peking Union Medical College Hospital with informed consent. The study was approved by the University of Kentucky IRB committee (IRB No. 11-0239-P6A).

### Whole genome sequencing of the “discovery set” samples

Whole genome sequencing was performed using genomic DNA from the seven individuals in the “discovery set” (Supplementary Table S1). Genomic DNA was isolated from either paraffin-embedded tissues (cases 1–3) or blood (cases 4–9). Sequencing libraries were constructed according to the standard protocol and hybridized to the surface of flow cells to form clusters in Illumina cBot, then sequenced on the Illumina HiSeq2000 (San Diego, CA) platform. Raw image files were processed by the Illumina Pipeline (version 1.3.4) for base-calling with default parameters. Finally, we obtained ~130 GB raw data (90-bp pair-end reads) for each sample with genomic coverage ranging from 27 to 40 folds, which is enough for variation identification.

### Alignment and variation identification

The sequencing reads were aligned to the University of California Santa Cruz human genome reference (assembly hg19) by the BWA (Burrows-Wheeler Aligner) alignment pipeline with default parameters^10^. Duplication rates of all samples were <3.6%, and duplication reads were marked by the Picard (MarkDuplicate) package. The BAM files derived from BWA alignment were then processed by the Genome Analysis ToolKit (GATK v1.6)^11^ to realign around known Insertion and Deletion (InDel) sites. All aligned reads were subjected to GATK Count Covariates based on known SNVs (dbSNP137), then base quality was recalibrated by GATK Table Recalibration.

The uniquely mapped reads were used for variation identification. GATK UnifiedGenotyper was used to identify genome-wide SNVs and InDels. Then FamSeq^41^ was used to revise SNVs of the family, and SNV sites shared by more than two family members were retained. As the available methods for identifying CNVs and SVs are not very accurate, we combined two different pipelines to do this. CNVs were called by ReadDepth^42^ and CNVnator^43^, and SVs were called by BreakDancer^44^ and Pindel^45^. Finally, we used Annovar^12^ to annotate all the variants.

### Linkage analysis and variant filtering

SNVs with quality score ≥ 30 that were included in the HapMap dataset were extracted to estimate the linkage region of this family by the Merline pipeline^46^. Regions with positive LOD scores were selected as candidate inherited regions.

SNVs in the positive LOD region with VAF (variant allele frequency) > 0.5% in the dbSNP137, HapMap (CEU) and 1000 Genomes project datasets (CEU) were excluded, and only non-synonymous, splicing variations were retained. SIFT^16^ and Polyphen2^17^ were used to predict the perniciousness of these variations, and SNVs predicted to be “Damage” or “Possible Damage” were reserved. Then, based on multiple inheritance models (recessive or dominant, homozygous or heterozygous), SNVs found in abnormal samples (SF-001M, SF-002G, and SF-002P; we treated SF-002P as a tumor case in our analysis) but not in normal samples (SF-002S) were considered candidate causative mutations.

Similarly, InDels in the positive LOD region with VAF > 0.5% in both the dbSNP137 and 1000 Genomes project datasets (CEU) or in the extended region of such variations were excluded. Only frameshift variations harbored in abnormal samples, but not in normal samples, were considered candidate causative mutations.

### Conservation analysis

Mutations at evolutionarily conserved regions are more likely to be functional and detrimental to organisms. We applied two methods to test the conservation of the discovered mutations: (a) we searched the phylop database and considered variations with a phylop score > 1 to be conserved sequences^47^; and (b) we used the Basic Local Alignment Search Tool (BLAST) to compare the local genome sequence around the variations with all other species’ sequences to assess the conservation properties of the candidate variations. Extended regions with different lengths (50, 100, 200, 500, and 1000 bp) around the candidate variations were extracted from the human genome sequence to test the conservation, and nearly all sequences could be mapped to more than one other species.

### Haplotype analysis

Because there are two genetically unrelated samples in the family (SF-001M and SF-002S), we grouped the samples into three subfamilies (the whole family, SF-001M and her children, and SF-002P & SF-002S and their children) when performing haplotype analysis to obtain more accurate haplotype information. Variations in the haplotype shared by more than two affected samples (SF-001M, SF-002G, and SF-002P) were considered to be the inherited genetic mutations, which are more detrimental.

### Target sequencing of the “validation set” samples

Genomic DNA were isolated from the 49 “validation set” samples to amplify the coding regions of the genes identified by PCR. The Wafergen (Fremont, CA) Smartchip (4-Primer Sequencer-Ready) was used to amplify the target regions and prepare the sequencing library. Then, the DNA library was sequenced on the Illumina Hiseq2000 platform with 500 × sequencing depth. After standard quality control, the sequencing reads were mapped to the genome by the Burrows-Wheeler Aligner (BWA) package. Mutations, including SNVs and InDels, were called for all samples by the GATK toolkit, as described previously. Among the mutations, somatic SNVs were called for the 19 pairs of normal-tumor cases by Varscan^48^, and InDels were detected by SAMtools^49^. Non-synonymous variants with low VAF in the dbSNP137 and 1000 Genomes datasets and predicted to be “Damage” or “Possible Damage” by SIFT and Polyphen2 were reserved as detrimental mutations.

### Comparative analysis of mutation frequencies

The International Cancer Genome Consortium (ICGC) database contains a comprehensive description of genomic mutations in 50 different tumor types and/or subtypes. We searched the database for mutations in the genes we identified by WGS and chose mutations with function effects annotated as “High” and “Low” to analyze the mutation frequencies of each gene in 19 tumor types and/or subtypes. Mutation frequency was calculated as the percentage of cases with mutations in the target genes.

### Protein–protein interaction network analysis

The PPi map of *H. sapiens* was downloaded from the Human Protein Reference Database (HPRD). The interaction network of the identified candidate genes was constructed by mapping them to the *H. sapiens* protein–protein interaction map. Then, the interaction network was visualized and modified by Cytoscape v2.8.3 (http://www.cytoscape.org).

### Molecular dynamics simulation analysis (MDSA)

Protein structures were predicted by the online I-TASSER method^50^ and then used for simulation analysis. The GROMACS 4.6.5 package^51^, Gromos96 (54a7) forcefield^52^, and tip3p water model^53^ were used for simulations. Each system was simulated under periodic boundary conditions in a dodecahedron box with a 1.0 Å edge length. Energy minimization for the solvated structures was performed using the Steepest Descent method and was carried out until the maximum force reached 100 KJ/mol/nm (Max.force < 100 KJ/mol/nm). The energy-minimized structures were subjected to position restrained dynamics simulation for 200 ps, keeping the whole protein molecule fixed and allowing only the water molecules to move and equilibrate. The temperature of the full system was maintained at 300 K by independently coupling the protein and the solvent to an external temperature bath with a coupling constant of 0.1 ps using a V-rescale thermostat. The pressure was maintained at 1 bar by coupling the system to an isotropic pressure bath using a coupling constant of 2 ps and Parrinello-Rahman’s barostat. The cut-off for an electrostatic interaction of Van dar Waal and coloumbic was defined as 0.1 nm radius. For long-range correction of the electrostatic interaction, the particle mesh Ewald (PME) method was used with Fourier spacing of 0.12 and PME interpolation order setting of 4. During the simulations, all bond lengths were constrained using the LINCS algorithm, and the SETTLE algorithm was used to constrain the geometry of the water molecules. All the MDSs ran for 20 ns with 2 fs time intervals. After the simulation was completed, the trajectory files were generated and analyzed by different GROMACS tools.

### Plasmids and western blot

The pC.HA-Vav2 (Plasmid #14554) and pcDNA3-Myc-IQGAP1 (Plasmid #30118) plasmids were purchased from Addgene. The plasmids expressing mutant VAV2 and IQGAP1 were derived from the WT plasmids through mutagenic PCR. Empty vectors and plasmids containing WT and mutant Coding sequences were transfected into HEK293 or HeLa cells, which were harvested for western blot 48 hours after transfection. Antibodies against HA tag (Santa Cruz, sc-7392), Myc (Sigma, C3956), ERK (Santa Cruz, sc-514302), phospho-ERK (Santa Cruz, sc-7383), AKT (Santa Cruz, sc-81434) and phospho-AKT (Santa Cruz, sc-81433) were used.

## Supplementary information

Supplementary figures and tables

## Supplementary information

**Supplementary Information** accompanies the paper at (10.1038/s41421-019-0126-6).
