# Author Correction: Identification of novel genetic variants predisposing to familial oral squamous cell carcinomas

**DOI:** 10.1038/s41421-020-00189-3

**Published:** 2020-07-14

**Authors:** Yaping Huang, Jizhi Zhao, Guogen Mao, Grace Sanghee Lee, Jia Zhang, Lijun Bi, Liya Gu, Zhijie Chang, Joseph Valentino, Guo-Min Li

**Affiliations:** 1 Department of Radiation Oncology, University of Texas Southwestern Medical Center, Dallas, TX 75390 USA; 2 Department of Stomatology, Peking Union Medical College Hospital, Beijing, 100730 China; 3 Department of Toxicology and Cancer Biology, University of Kentucky College of Medicine, Lexington, KY 40536 USA; 4 Insititute of Biophysics, Chinese Academy of Sciences, Beijing, 100101 China; 5 Department of Basic Medical Sciences, Tsinghua University School of Medicine, Beijing, 100084 China; 6 Department of Otolaryngology, Head & Neck Surgery, University of Kentucky College of Medicine, Lexington, KY 40536 USA

**Keywords:** Cancer genetics, Oral cancer, Oncogenes

Correction to: *Cell Discovery* (2019) 5:57

10.1038/s41421-019-0126-6 Published online 26 November 2019

In the original publication of this article^1^, one of the supporting sources was missing in the Acknowledgements part. The correct Acknowledgements appear below:
