# Supplementary material for: Identification of novel genetic variants predisposing to familial oral squamous cell carcinomas
Source: Cell Discov. 2019 Nov 26;5:57. doi: 10.1038/s41421-019-0126-6 (PMC6877579; doi:10.1038/s41421-019-0126-6)
Supplement: Supplementary file 1 — Supplementary figures and tables [file 41421_2019_126_MOESM1_ESM.pdf]

Supplementary Information

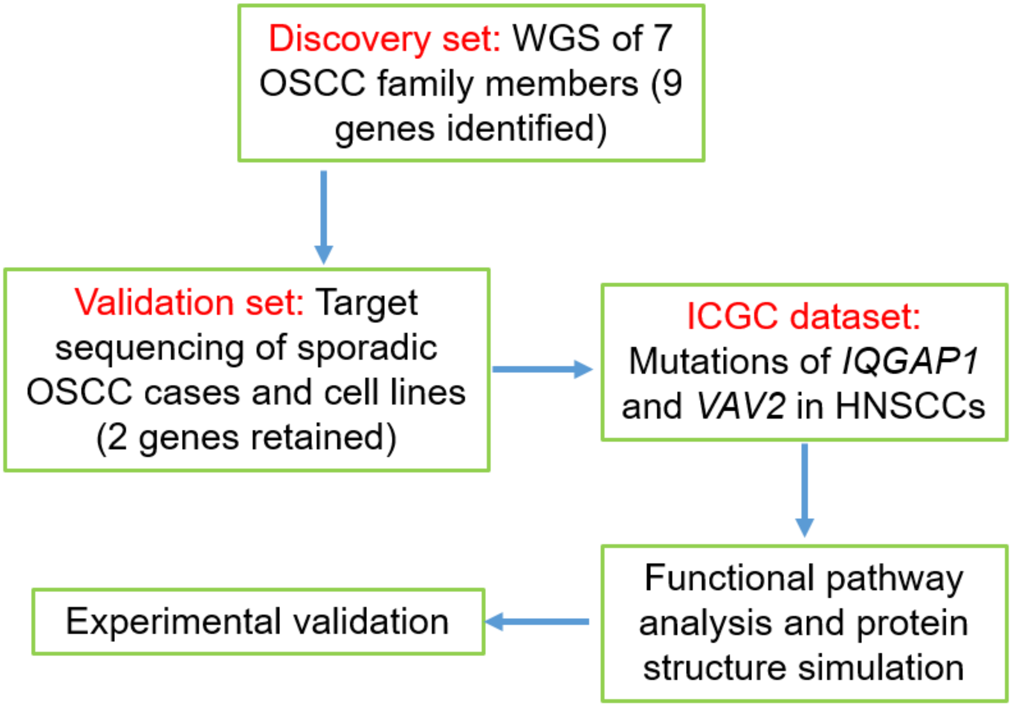

Supplementary Fig. S1. The flow chart of the whole study.

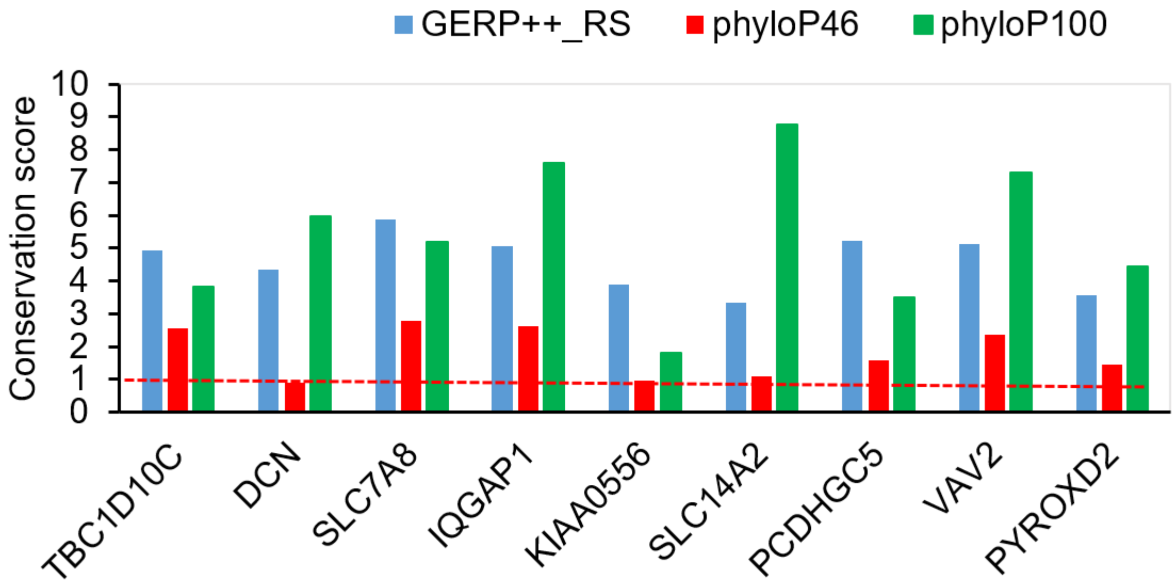

Supplementary Fig. S2. Sequence conservation analysis of the identified variants. The conservation scores predicted by three independent datasets (GERP++_RS, blue; phyloP46, orange; phyloP100, grey) are shown (Y axis) for each of the identified genes (X axis). All scores are greater than one, indicating that all the variants are relatively conserved across species.

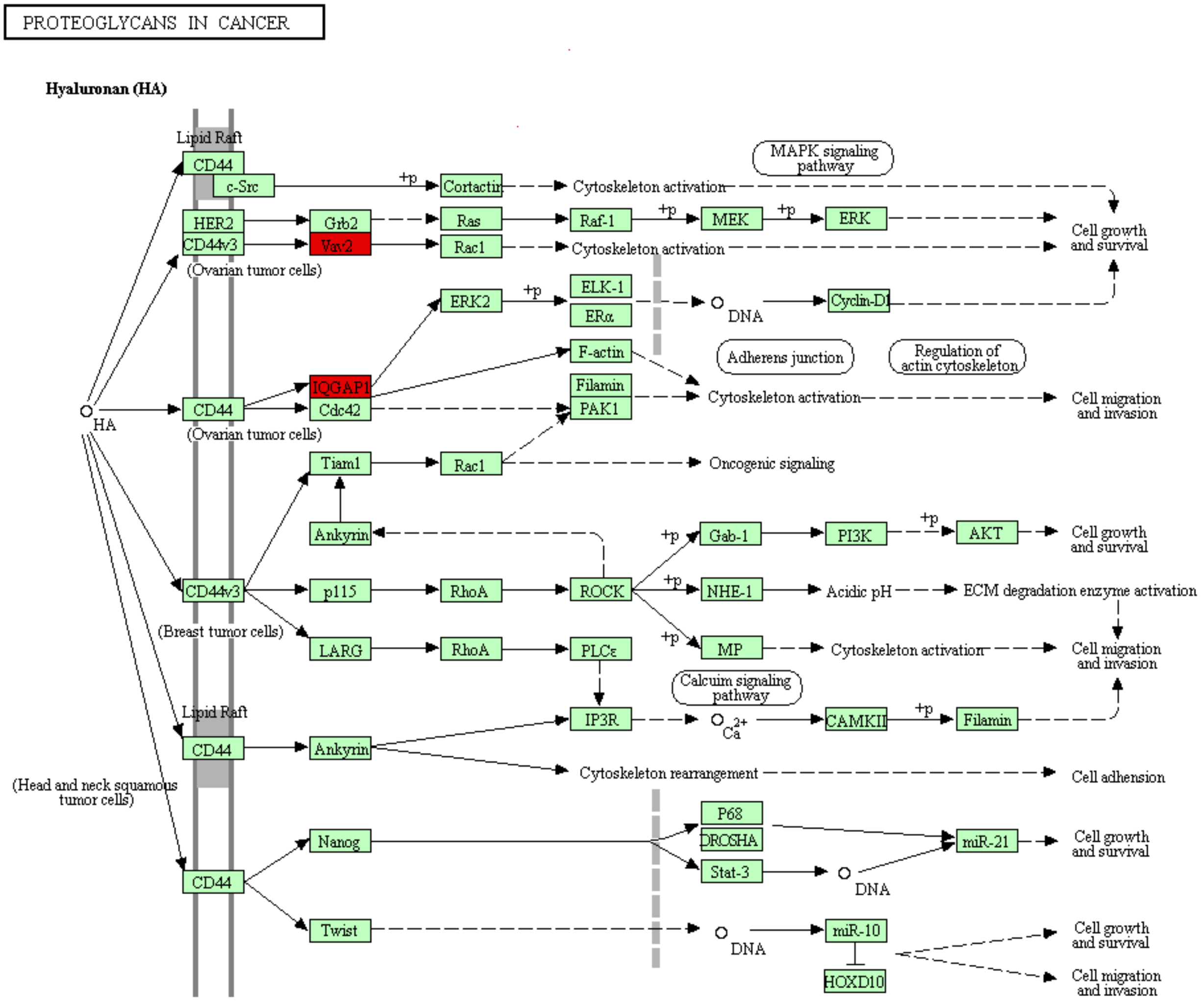

Supplementary Fig. S3. Picture of part of the KEGG pathway “Proteoglycans in cancer” (hsa05205). VAV2 and IQGAP1 are highlighted in red.

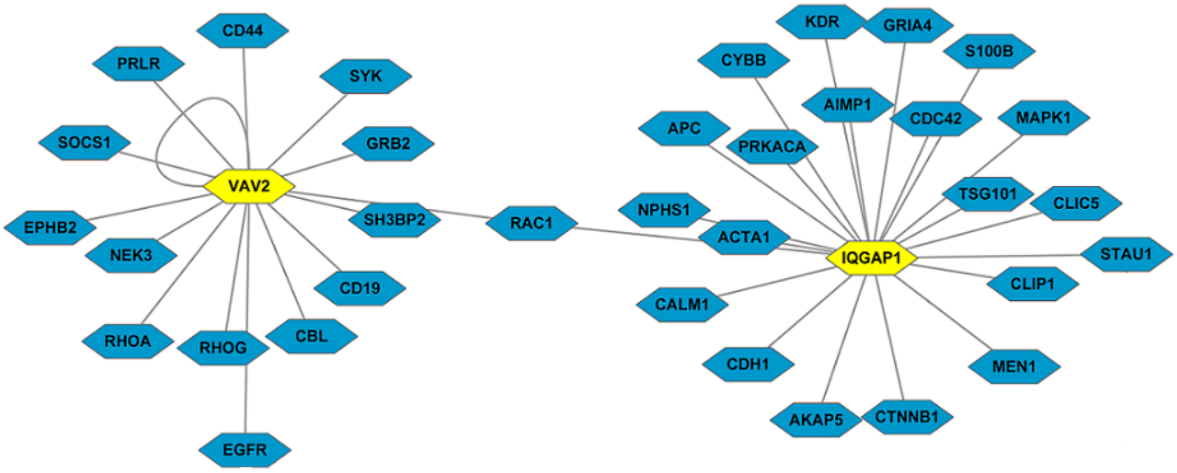

Supplementary Fig. S4. Protein-protein interaction network of the proteins encoded by the candidate genes. In the network, the identified genes (yellow node) are in the center, and the interacting ones (blue node) are outside. Each edge links one yellow node and one blue node, which indicates an interaction between the two proteins.

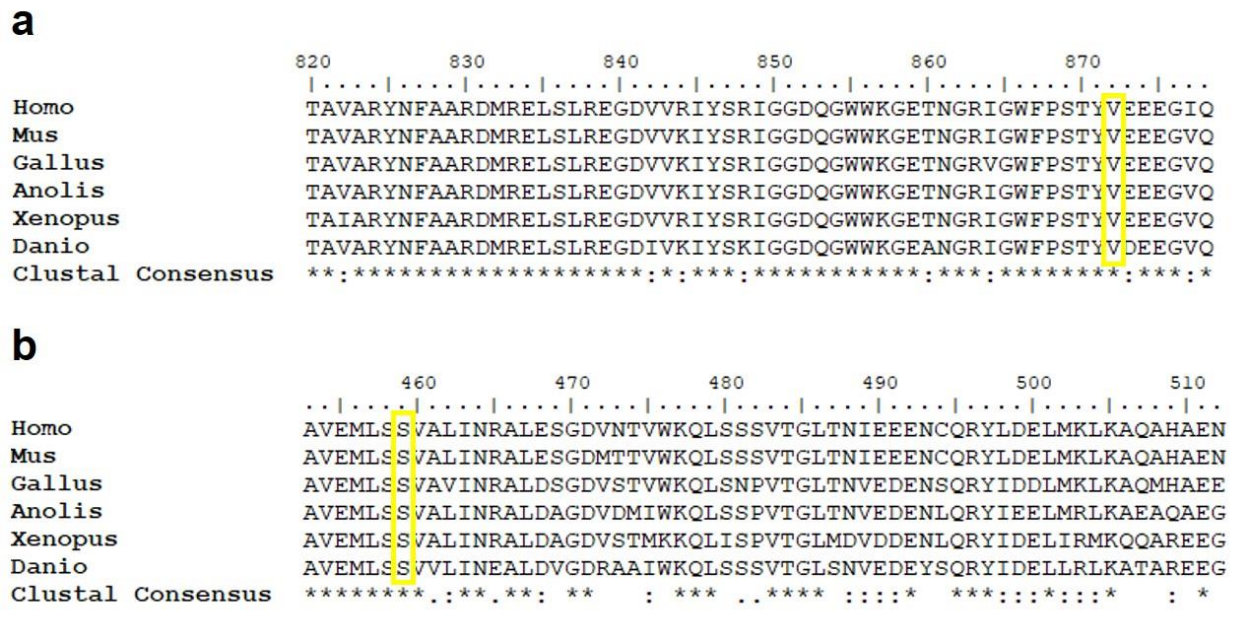

Supplementary Fig. S5. Clustal omega multi-sequence alignments of VAV2 and IQGAP1 amino acid sequences across species. **(a)** Multi-sequence alignments of VAV2 amino acid sequences across vertebrates. The mutated amino acid Val872 is indicated by the yellow box. **(b)** Multi-sequence alignments of IQGAP1 amino acid sequences across vertebrates. The mutated amino acid Ser459 is highlighted by the yellow box.

Supplementary Table S1. The detail information of the family members

Sample ID	Sample name	Sex	Age	Oral cancer	Mortality	Tobacco or alcohol user	Other notes
1	SF-001M	F	-	Yes	died	No	Type I diabetes
2	-	M	-	Yes	died	No	-
3	SF-002G	F	-	Yes	died	No	-
4	SF-002P	M	61	Yes	alive	No	Type I diabetes since 44 years old
5	SF-003S	F	27	No	alive	No	Type I diabetes since 9 years old
6	SF-003J	M	31	No	alive	No	-
7	SF-003C	M	31	No	alive	No	-
8	-	M	-	No	died	-	-
9	SF-002S	F	-	No	alive	No	-

Supplementary Table S2. Statistics for whole genome sequencing coverage and depth

Sample	SF-002G	SF-001M	SF-003C	SF-002P	SF-003S	SF-003J	SF-002S
GC content rate	39.47%	38.51%	41.10%	41.76%	41.01%	41.50%	40.31%
mismatch rate	0.53%	0.68%	0.49%	0.48%	0.56%	0.50%	0.35%
reads length(bp)	90	90	90	90	90	90	90
Coverage rate	99.91%	99.91%	99.92%	99.93%	99.93%	99.93%	99.92%
Coverage >= 4X	99.64%	99.41%	99.84%	99.84%	99.79%	99.84%	99.90%
Coverage >= 8X	99.07%	97.53%	99.66%	99.64%	99.60%	99.66%	99.69%
Coverage >= 10X	98.56%	95.60%	99.48%	99.41%	99.45%	99.47%	99.40%
Coverage >= 20X	91.19%	73.18%	95.80%	94.82%	97.12%	95.50%	96.00%
Mean Depth	37.11	27.12	41.80	40.54	40.83	40.74	41.84
Coverage of chrX	99.92%	99.93%	99.89%	99.91%	99.94%	99.90%	99.88%
Coverage of chrY	18.00%	17.92%	99.17%	99.18%	17.45%	99.17%	14.94%
Duplication rate	3.31%	3.58%	1.79%	1.93%	1.67%	1.89%	5.62%

Supplementary Table S3. Statistics for all SNVs identified in each sample

Sample	SF-002G	SF-001M	SF-003C	SF-002P	SF-003S	SF-003J	SF-002S
downstream	22,273	21,014	23,028	22,964	22,901	23,039	23,688
exonic	426	375	445	456	462	447	414
nonsense	70	57	81	84	73	81	72
readthrough	12	11	8	12	11	8	11
synonymous	11,130	10,005	11,679	11,587	11,654	11,672	11,658
missense	10,089	8,955	10,737	10,564	10,652	10,702	10,756
exonic;splicing	299	271	290	298	288	293	277
intergenic	2,400,147	2,312,941	2,437,405	2,442,237	2,459,668	2,431,886	2,487,889
intronic	1,292,182	1,231,568	1,311,612	1,310,792	1,323,570	1,310,525	1,351,868
ncRNA_exonic	8,152	7,172	8,421	8,337	8,652	8,423	8,532
ncRNA_intronic	99,280	93,026	99,491	99,426	99,663	99,250	103,437
ncRNA_splicing	43	43	45	43	45	41	47
ncRNA_UTR3	425	383	420	406	404	420	462
ncRNA_UTR5	73	48	78	71	62	74	65
splicing	103	93	111	116	111	107	116
upstream	20,021	17,877	22,780	22,504	22,564	22,641	22,553
upstream;downstream	609	574	748	698	683	744	687
UTR3	24,430	23,246	24,496	24,618	24,714	24,513	25,006
UTR5	4,088	3,180	4,999	4,987	4,973	5,026	4,852
UTR5;UTR3	10	9	9	10	8	9	11
All	3,893,563	3,730,577	3,956,593	3,959,912	3,990,870	3,949,608	4,052,401

Supplementary Table S4. Statistics for all InDels identified in each sample

Sample	SF-002G	SF-001M	SF-003C	SF-002P	SF-003S	SF-003J	SF-002S
downstream	3,904	3,190	4,375	4,293	4,239	4,361	4,715
exonic;splicing	14	8	15	18	15	16	14
intergenic	348,189	298,575	375,027	372,950	366,770	370,371	400,442
intronic	207,673	173,238	225,513	223,547	220,243	223,915	243,393
ncRNA_exonic	878	692	932	907	934	915	977
ncRNA_intronic	14,951	12,298	15,988	15,923	15,324	15,740	17,413
ncRNA_splicing	6	5	10	11	7	8	3
ncRNA_UTR3	63	58	84	79	79	87	86
ncRNA_UTR5	5	3	8	8	9	7	9
splicing	73	55	72	71	74	74	74
upstream	2,887	2,218	3,850	3,788	3,705	3,886	3,906
upstream;downstream	97	77	117	119	102	117	115
UTR3	4,814	4,196	5,128	5,003	5,044	5,089	5,418
UTR5	375	229	650	666	615	665	529
UTR5;UTR3	2	3	2	3	2	3	1
frameshift	112	87	150	139	134	149	148
nonframeshift	199	137	275	302	273	277	259
missense	6	4	10	10	7	9	4
nonsense	3	3	5	3	6	5	6
readthrough	0	0	0	0	1	0	1
synonymous	1	1	1	1	1	1	4
exonic	63	60	79	77	75	80	73
All	584,315	495,137	632,291	627,918	617,659	625,775	677,590

Supplementary Table S5. Statistics for all CNVs identified in each sample

Sample	SF-002G	SF-001M	SF-003C	SF-002P	SF-003S	SF-003J	SF-002S
downstream	3	2	5	6	6	8	4
exonic	90	84	152	162	131	164	137
intergenic	438	460	784	761	715	752	729
intronic	92	87	207	206	201	226	226
ncRNA_exonic	47	47	60	69	57	61	58
ncRNA_intronic	3	6	23	24	22	26	18
splicing	6	1	13	15	11	16	9
upstream	1	2	8	8	10	11	9
upstream;downstream	1	0	0	0	0	0	0
UTR3	0	0	0	1	0	0	5
UTR5	0	0	0	0	0	0	1
All	681	689	1,252	1,252	1,153	1,264	1,196

Supplementary Table S6. Statistics for all SVs identified in each sample

Sample	SF-002G	SF-001M	SF-003C	SF-002P	SF-003S	SF-003J	SF-002S
UTR3	2	0	2	0	0	11	1
UTR5	0	0	2	0	0	6	2
downstream	0	0	3	1	2	18	2
exonic	129	66	438	153	174	945	327
intergenic	324	299	1,083	403	556	2,833	831
intronic	26	23	233	91	122	652	171
ncRNA_exonic	33	11	106	29	35	398	57
ncRNA_intronic	1	0	9	2	3	17	7
splicing	0	0	3	0	0	9	4
upstream	0	0	6	2	1	17	5
Total	515	399	1,885	681	893	4,906	1,407

Supplementary Table S7. The filtration algorithm for identification of hereditary SNVs and Indels

Filtration algorithm	SF-001G	SF-002M	SF-003C	SF-002P	SF-003S	SF-003J	SF-002S
Exclude SNVs with low quality	3,348,181	3,172,066	3,340,598	3,343,787	3,376,830	3,340,376	3,374,025
Exclude SNVs in HapMap (VAF>0.5%)	1,900,956	1,787,710	1,866,082	1,871,055	1,901,158	1,865,324	1,893,571
Exclude common SNVs in 1000 Genomes (VAF>0.5%)	309,771	290,785	268,190	274,344	295,094	267,768	292,363
Exclude SNVs in dbSNP	156,721	148,507	139,768	146,020	147,996	139,529	144,653
Non-synonymous	282	258	230	262	236	230	203
Disease inheritance mode analysis	59	59	37	59	23	37	0
Function prediction	20	20	11	20	8	11	0
Haplotype analysis	10	10	6	10	3	6	0

Supplementary Table S8. Clinical information of the sporadic OSCCs

Tumor sample	Normal control	Age	Sex	Pathological diagnosis	Life style (Tobacco / alcohol user)	HPV status	Biopsy done	cancer family history
S1	N1	48	M	Grade I & II tongue squamous cell carcinoma	Y/Y	-	Y	N
S2	N2	62	M	Tongue squamous cell chronic inflammation, anabrosis	N/N	-	N	N
S3	N3	-	M	-	-	-	-	-
S4	N4	55	F	Grade I tongue squamous cell carcinoma	N/N	-	Y	N
S7	N7	76	F	Tongue squamous cell carcinoma	N/N	-	Y	N
S8	-	72	M	Grade II tongue squamous cell carcinoma	Y/Y	-	Y	N
S10	N10	51	M	Grade I tongue squamous cell carcinoma, lymphatic metastasis	Y/Y	-	Y	N
S12	N12	54	F	Grade II tongue squamous cell carcinoma	N/N	-	Y	N
S13	N13	48	M	Tongue squamous cell carcinoma	N/N	-	Y	N
S14	N14	57	M	Grade II tongue squamous cell carcinoma	N/Y	-	Y	N
S15	-	56	M	Grade I tongue squamous cell carcinoma	Y/Y	-	N	N
S16	N16	61	M	Grade I tongue squamous cell carcinoma	N/N	-	N	N
S17	N17	38	F	Grade I tongue squamous cell carcinoma	N/N	-	N	N
S19	N19	67	M	Grade II tongue squamous cell carcinoma	N/N	-	N	N
S20	N20	60	M	Grade I tongue squamous cell carcinoma	N/N	-	Y	N
S21	N21	47	M	Grade I tongue squamous cell carcinoma	Y/Y	-	N	N
S22	N22	51	M	Grade II tongue squamous cell carcinoma, lymphatic metastasis	Y/Y	-	Y	N
S23	N23	-	F	-	-	-	-	-
S24	N24	53	M	Grade II tongue squamous cell carcinoma	N/N	-	N	N
S25	-	63	M	Tongue squamous cell carcinoma	Y/Y	-	Y	N
S26	-	74	F	Hard palate squamous cell carcinoma	N/N	-	Y	N
S27	N27	61	M	Grade I tongue squamous cell carcinoma, lymphatic metastasis	Y/Y	-	Y	N
S28	-	53	M	Grade I tongue squamous cell carcinoma	Y/Y	-	Y	N
S29	N29	57	M	Tongue squamous cell carcinoma	N/N	-	Y	N
S30	-	-	-	Tongue squamous cell carcinoma	N/N	-	-	-
S31	-	-	-	Tongue squamous cell carcinoma	N/N	-	-	-

Supplementary Table S9. Summary of SVs in familial OSCCs

Gene involved	SV type	Chr	Start	End	Annotation
Almost the entire chromosome	DEL	chr1	725430	224199634	not confident
CDC123, RP11-186N15.3, CAMK1D	DEL	chr10	12273782	12434907	gene region truncation

Supplementary Table S10. Summary of CNVs in familial OSCCs

Genome element	Proximal gene	CNV type	Chr	Start	End
intergenic	AC142119.1;SNORA40	deletion	chr2	16270801	16274400
intron	AC007392.3	deletion	chr2	66855601	66870900
intergenic	RP11-317G22.2;CWH43	deletion	chr4	48943801	48953400
intergenic	U6;end	deletion	chr16	90294701	90354800
intergenic	ADAMTS10;ACTL9	deletion	chr19	8687201	8737200
intergenic	SNORA70;IGHV1OR21-1	deletion	chr21	10647801	10697900
intergenic	LIMK1;EIF4H	deletion	chr7	73549701	73551000
intergenic	DDX11L16;end	deletion	chrX	1.55E+08	1.55E+08
